# Educational attainment, body mass index, and smoking as mediators in kidney disease risk: a two-step Mendelian randomization study

**DOI:** 10.1080/0886022X.2025.2476051

**Published:** 2025-03-11

**Authors:** Lei Zhang, Baiyu Feng, Zhiwen Liu, Yu Liu

**Affiliations:** Department of Nephrology, The Second Xiangya Hospital at Central South University, Hunan Key Laboratory of Kidney Disease and Blood Purification, Changsha, Hunan, China

**Keywords:** Educational attainment, Mendelian randomization, mediation analysis, modifiable risk factors, kidney disease

## Abstract

**Background:**

Educational attainment (EA) has been linked to various health outcomes, including kidney disease (KD). However, the underlying mechanisms remain unclear. This study aims to assess the causal relationship between EA and KD and quantify the mediation effects of modifiable risk factors using a Mendelian randomization (MR) approach.

**Methods:**

We performed a two-sample MR analysis utilizing summary statistics from large-scale European genome-wide association studies (GWAS). EA (NGWAS = 766,345) was used as the exposure, and KD (Ncase/Ncontrol= 5,951/212,871) was the outcome. A two-step MR method was applied to identify and quantify the mediation effects of 24 candidate risk factors.

**Results:**

Each additional 4.2 years of genetically predicted EA was associated with a 32% reduced risk of KD (odds ratio [OR] 0.68; 95% confidence interval [CI] 0.56, 0.83). Among the 24 candidate risk factors, body mass index (BMI) mediated 21.8% of this protective effect, while smoking heaviness mediated 18.7%.

**Conclusions:**

This study provides robust evidence that EA exerts a protective effect against KD, partially mediated by BMI and smoking. These findings highlight the potential for targeted public health interventions aimed at mitigating obesity and smoking-related risks to reduce KD incidence, particularly among individuals with lower educational attainment.

## Introduction

Kidney disease (KD) encompasses a variety of disorders that impair kidney function, with even mild abnormalities increasing the risk of complications and mortality [1]. It is primarily classified into acute kidney injury (AKI), which is characterized by a rapid decline in kidney function, and chronic kidney disease (CKD), where dysfunction persists for more than three months [2]. AKI can progress to CKD, and conversely, CKD increases the likelihood of experiencing AKI. The incidence of CKD is rising, likely driven by the increasing prevalence of risk factors such as obesity and diabetes mellitus. In 2017, an estimated 843.6 million people worldwide were affected by CKD [3]. Recent studies have shown that the burden of CKD was concentrated in areas with relatively low socio-demographic index (SDI), most notably in Oceania, Sub-Saharan Africa, and Latin America [4]. A meta-analysis including 154 studies showed that the incidence rate of AKI was higher in Southern Europe (31.5%) and South America (29.6%) compared to the other regions with relatively higher SDI included in the study [5]. Also, studies indicated that a higher risk of AKI was noted in comparatively deprived areas, and renal failure was linked to low socioeconomic status [6–8]. Other recent observational studies also revealed that higher education, a reliable indicator of socioeconomic position [9], was associated with reduced incidences of CKD in the general population and type 2 diabetes patients, as well as decreased risk of end-stage renal disease in type 1 diabetes patients.

Previous observational studies indicated that the waist-to-hip ratio (WHR), hypertension, body mass index (BMI), smoking, and potassium excretion are likely associated with the relationship between education and CKD [10–12]. However, the conclusion based on these observational studies was unreliable due to the easy influence of confounders and reverse causation. Therefore, a method free from the biases of traditional observational study is required to answer the question of what the mediators are and what their pathway is in the association between education and renal failure.

Mendelian randomization (MR) is a technique that utilizes genetic variants identified in genome-wide association studies (GWAS) as instrumental variables to deduce the causal association between exposure and outcome. As allelic variants are randomly allocated during conception, MR can emulate randomized controlled studies and minimize bias [13]. Albeit, previous MR studies showed the association between some of the renal risk factors (e.g., blood lipids, obesity, blood pressure) and CKD [14–16]. And, a recent MR study revealed that educational attainment (measured by years of schooling) had a protective effect on CKD [17]. The relationship between educational attainment and KD, and the potential mediation by modifiable renal risk factors, remains unclear. To address this, we utilized univariable MR to examine the link between education and renal failure, followed by a two-step MR analysis to determine the contribution of these risk factors to this connection. The potential mediators in the two-step analysis, collected from published literature, show a demonstrated link with KD. Our findings suggest that implementing population-level interventions to address modifiable risk factors such as WHR, BMI, smoking, and systolic blood pressure (SBP) could substantially reduce the risk of renal failure, particularly among individuals with lower educational levels. This research can be instrumental in helping those with limited educational backgrounds lower their risk of renal failure and improve overall renal health.

## Materials and methods

### Overall study design

**Figure 1.**
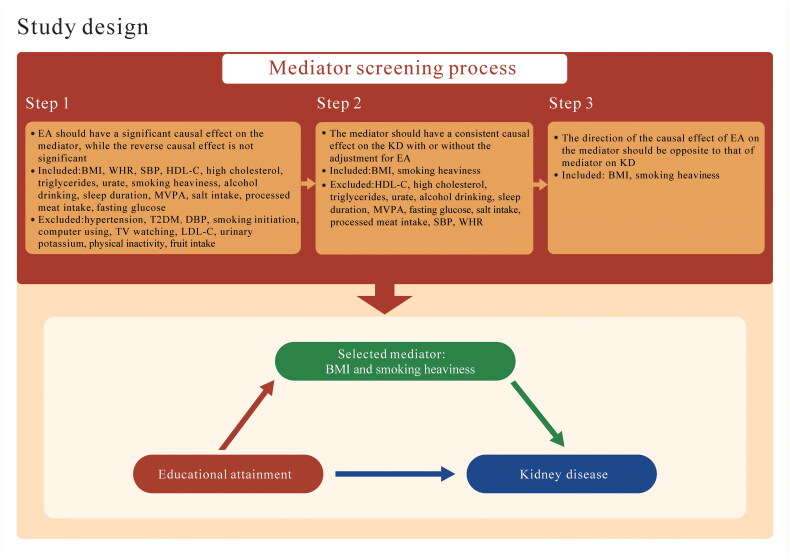
The flow of the study. EA, educational attainment; KD; BMI, body mass index; WHR waist-to-hip ratio; SBP, systolic blood pressure; DBP, diastolic blood pressure; HDL-C, high-density lipoprotein cholesterol; MVPA, moderate to vigorous physical activity; LDL-C, high-density lipoprotein; cholesterol; TV, television.

The Mendelian randomization analysis relies on three fundamental assumptions: (1) the genetic variants used as instruments must have a significant correlation with the exposure. (2) the instrumental variables should not be linked to confounding factors that influence both the exposure and outcome. (3) the genetic variants should only relate to the outcome through the exposure [18]. To assess the causal effect of EA on KD, we conducted univariable MR analysis, which revealed a negative association between EA and KD. We then screened for candidate mediators that could contribute to the protective effect of EA on KD. The screening process consisted of three steps: (1) we excluded mediators that showed bidirectional associations with EA, as this could complicate the interpretation of the mediation analysis [19]. (2) We removed mediators that exhibited inconsistent causal effects on KD, either before or after adjusting for EA. (3) Finally, we selected mediators with opposite causal effects, meaning the effect of EA on the mediator and the effect of the mediator on KD should operate in opposite directions. To assess the mediation effect of a single risk factor or a combination of multiple risk factors, we applied a two-step Mendelian randomization (MR) approach (Figure 1). For example, urate passed the first selection step because educational attainment was causally associated with urate, while no significant association was found in the reverse MR analysis, indicating that the relationship between educational attainment and urate is unidirectional, from EA to urate. However, in the second step, we found that urate was not causally associated with KD, whether or not EA was adjusted for, so it was excluded from further analysis.

### Ethics

The original studies that are included in this MR analysis had ethical approval and informed consent. Summary-level statistics used in the current study are publicly available, thus no further ethical approval was required.

### Data resources and instrumental variable selection

We obtained instrumental variables from GWAS data of each phenotype. GWAS of educational attainment (EA) was a meta-analysis including 1,131,881 European individuals conducted by the Social Science Genetic Association Consortium (SSGAC) (Table 1) [20]. However, the summary-level data of EA included in this study only covered 766,345 samples since the participants from 23andMe consortium were excluded from publicly accessible database. Since FinnGen does not provide a GWAS definition that directly corresponds to KD, we used ‘renal failure’ as the outcome, which encompasses the following conditions: acute renal failure, unspecified kidney failure, CKD, and dialysis. The outcome GWAS from FinnGen biobank consisted of 218,792 participants of European ancestry (5951 cases and 212871 controls) [21].

**Table 1.** A summary of GWAS data used in this MR study.

Phenotype	Unit	Sample size	Ancestry	Consortium	Author	Year of publication
Educational attainment	SD (4.2 years)	1,131,881	European	SSGAC	Lee et al.	2018
Educational attainment	SD (3.71 years)	293,723	European	SSGAC	Okbay et al.	2016
RF	Event	212,841	European	FinnGen	Kurki et al.	2022
BMI	SD (4.7 kg/m2)	681,275	European	GIANT	Yengo et al.	2018
WHR	SD (0.09)	212,244	European	GIANT	Shungin et al.	2015
Hypertension	Event	337,159	European	UK Biobank	Neale lab	2017
Fasting glucose	SD (0.73 mmol/L)	58,074	European	MAGIC	Scott et al.	2012
Diabetes	Event	461,578	European	UK Biobank	Ben et al.	2018
SBP	SD	436,419	European	UK Biobank	Ben et al.	2018
DBP	SD	436,424	European	UK Biobank	Ben et al.	2018
LDL-C	SD (0.87 mmol/L)	440,546	European	UK Biobank	Richardson et al.	2020
HDL-C	SD (0.38 mmol/L)	403,943	European	UK Biobank	Richardson et al.	2020
High cholesterol	SD	337,159	European	UK Biobank	Neale lab	2017
Triglycerides	SD	441,016	European	UK Biobank	Richardson et al.	2020
Urate	SD	343,836	European	UK Biobank	Neale lab	2018
Smoking initiation	Event	607291	European	GSCAN	Liu et al.	2019
Smoking heaviness	SD (8 cigarettes/d)	337334	European	GSCAN	Liu et al.	2019
Computer using	SD	360,895	European	UK Biobank	Ben et al.	2018
TV watching	SD	437,887	European	UK Biobank	Ben et al.	2018
Alcohol drinking	SD	336,965	European	UK Biobank	Neale lab	2017
Sleep duration	SD	460,099	European	UK Biobank	Ben et al.	2018
MVPA	SD (2084 MET-min/wk)	377 234	European	UK Biobank	Klimentidis et al.	2018
Physical inactivity	SD	460,376	European	UK Biobank	Ben et al.	2018
Potassium in urine	SD	326,816	European	UK Biobank	Hemani et al.	2017
Salt intake	SD	462,630	European	UK Biobank	Ben et al.	2018
Processed meat intake	SD	461,981	European	UK Biobank	Ben et al.	2018
Fruit intake	SD	446,462	European	UK Biobank	Ben et al.	2018

MR, Mendelian randomization; RF, renal failure; BMI, body mass index; MVPA, moderate to vigorous physical activity; WHR, waist-to-hip ratio; SBP, systolic blood pressure; SSGAC, Social Science Genetic Association Consortium; DBP, diastolic blood pressure; HDL-C, high-density lipoprotein cholesterol; TV, television; SD, standard deviation; GIANT, Genetic Investigation of Anthropometric Traits; LDL-C, high-density lipoprotein; GSCAN, GWAS & Sequencing Consortium of Alcohol and Nicotine use; MAGIC, Meta-Analyses of Glucose and Insulin-related traits Consortium; ICBP, International Consortium of Blood Pressure.

We chose a total of 24 candidate mediators based on published literature for the mediation analysis, and their relationship with KD was listed in Table S1. Summary information of mediators, including BMI [22], WHR [23], hypertension, fasting glucose [24], smoking initiation [25], smoking heaviness, diabetes, SBP, DBP, LDL-C [26], HDL-C, triglycerides, high cholesterol, urate, TV-watching, computer using, MVPA [27], physical inactivity, alcohol consumption, sleep duration, urinary potassium [28], salt intake, fruit intake and processed meat intake were also listed in Table 1. All of the relative information is of European descent.

We selected single-nucleotide polymorphisms (SNPs) as independent instrumental variables based on strict criteria (*p* < 5 × 10^−8^, r^2^ < 0.001, and clumping distance > 1Mb) and removed palindromic IVs [29]. In cases where SNPs were missing in the outcome, we utilized proxy SNPs in linkage disequilibrium (r^2^ > 0.9). We used F-statistics to evaluate the strength of instrumental variables and exposure in our UVMR analysis. The F value can be calculated with the formula: F = (N–K–1)/K × R^2^/(1–R^2^) [30]. This statistic was calculated based on sample size (N), number of selected SNPs (K), and the proportion of variance of the exposure explained by SNPs. R^2^ was calculated by 2 × EAF×(1-EAF)×beta^2^, where beta means the effect of SNP on the exposure, and EAF represents the effect allele frequency [31]. F-statistics greater than 10 indicate unlikely weak instrument bias [32].

### Statistical analysis

The inverse variance weighted (IVW) method was used as the primary analysis in both univariable and multivariable Mendelian randomization (UVMR and MVMR) analyses. IVW uses meta-analysis to combine the Wald estimate of each SNP, resulting in an unbiased causal estimation when pleiotropy is balanced.

We performed two-step MR analyses to calculate the mediation effect of each individual mediator in the relationship between EA and KD [33]. The product of coefficients method was employed for each individual mediator. First, we analyzed the effect of EA on the mediator. Second, we used IVs for the mediator to estimate the causal association between the mediator and KD after adjusting for EA. The indirect effect mediated by the mediator was then calculated by multiplying the causal effect from both steps. To calculate the combined mediation effect of multiple mediators, we used the following method. The indirect effect was the residual of the total effect of EA on KD in UVMR analysis and the direct effect of EA on KD in MVMR analysis which included both EA and multiple mediators. To calculate the proportion of the mediation effect of individual mediator or multiple mediators, we divided their indirect effect by the total effect. We estimated standard errors using the delta method [34].

### Sensitivity analyses

In addition to IVW, other methods like MR-Egger, weighted median, Radial MR, and Mendelian randomization pleiotropy residual sum and outlier (MR-PRESSO) were also used in UVMR analysis to confirm causal estimates. For MVMR analysis, MVMR-Egger was further utilized. When the instrument strength independent of direct impact (InSIDE) assumption is still valid, the MR-Egger can evaluate directional pleiotropy *via* the intercept of its regression model and provide reliable estimates of causal effect [35]. If more than 50% of the IVs included in the study are valid, the weighted median method yields a consistent estimate [36]. MR-PRESSO could identify outliers with potential horizontal pleiotropy and reevaluate the causal effect after removing those outlying SNPs [37]. MR-Egger, Radial MR, and MR-PRESSO tested the horizontal pleiotropy in the UVMR analysis, and the heterogeneity of IVs was evaluated using Cochran’s Q statistics.

To ensure the validity of causal associations, we only accepted IVW estimates that aligned in direction and significance with at least one method in the sensitivity analysis and exhibited no pleiotropy. (p for MR-Egger intercept > 0.05). We conducted a sensitivity analysis to assess potential bias from overlapping samples, as the GWAS meta-analyses on education and some mediators included UK Biobank participants. For this, we used an earlier GWAS of educational attainment [38] that excluded UK Biobank participants, providing a comparison to the main analysis of the relationship between EA and mediators. Additionally, the impact of sample overlap on the main analyses, which included the original EA GWAS, was further assessed using online tools [39].

Results were presented as odds ratio (OR), β coefficient, and proportion with corresponding 95% confidence interval (CI). *p* < 0.05 was used as the significance threshold. All analyses were performed in R (version 4.13) using ‘TwoSampleMR’, ‘MendelianRandomization’, ‘MVMR’, ‘RadialMR’ and ‘MRPRESSO’ packages.

## Results

### Educational attainment had a protective effect on renal failure

**Figure 2.**
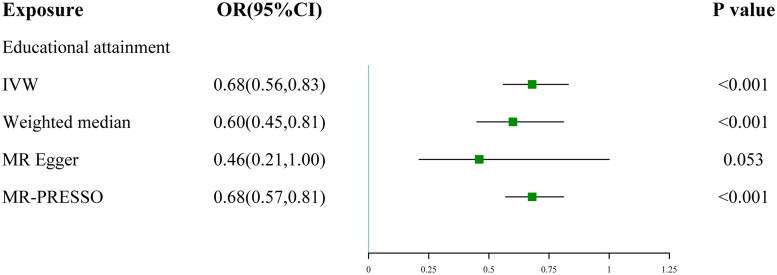
The forest plot of education’s effect on kidney disease using different MR methods. OR, odds ratio; CI, confidence interval; IVW, inverse variance weighted; MR, mendelian randomization; MR-PRESSO, mendelian randomization pleiotropy residual sum and outlier.

A total of 257 genetic variants of EA were used in the UVMR analysis, explaining 1.84% of its variance, and the general F statistics is 56, and the IVs were listed in Table S14. The causal estimate indicated that EA had a protective effect on KD (OR 0.68; 95% CI 0.56, 0.83). The IVW result was further validated in the sensitivity analysis evaluated by other two MR models (Figure 2): MR-Egger, Radial MR as well as MR-PRESSO results showed no evidence of pleiotropy (*p* > 0.05) (Tables S3, S4, and S12). Heterogeneity was not detected in Cochran’s Q analysis (Table S2). A previous study has shown that cognition may be both phenotypically and genetically associated with EA [40]. To account for the potential influence of cognition, we conducted MVMR analysis, adjusting for cognitive performance. Our analysis revealed that EA remains negatively associated with KD, regardless of whether cognition is adjusted for (p for IVW < 0.05) (Table S15).

### Educational attainment was negatively associated with BMI, WHR, smoking heaviness, and SBP

**Figure 3.**
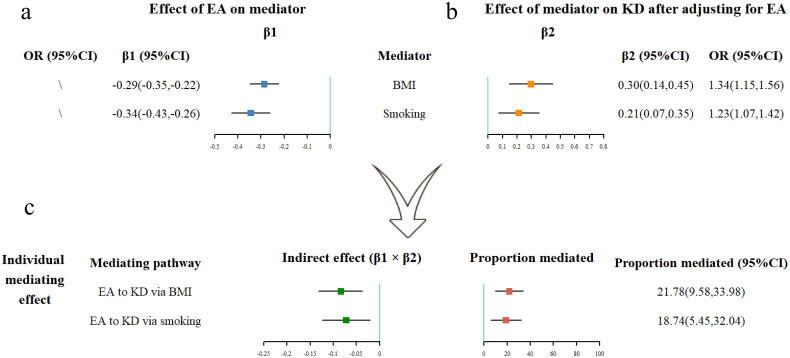
(a) The forest plot of educational attainment’s effect on each mediator. (b) The forest plot of each mediator’s effect on KD after adjusting for education. (c) The forest plot of the indirect effect of each mediator and the mediated proportion of each mediator. OR, odds ratio; CI, confidence interval; EA, educational attainment; KD, kidney disease; BMI, body mass index.

Four causal mediators were selected from 24 risk factors through the screening process. Each standard deviation (SD) (4.2 years) higher in educational attainment was associated with lower BMI (β −0.29; 95% CI −0.35, −0.22), lower WHR (β −0.23; 95% CI −0.29, −0.18), lower SBP (β −0.13; 95% CI −0.17, −0.09), and lower smoking heaviness (β −0.34; 95% CI −0.43, −0.26) (Figure 3). There was no indication of pleiotropy according to the MR-Egger model, although significant heterogeneity was found in four UVMR analyses (Table S3). Despite the fact that outlying IVs were detected by MR-PRESSO and Radial MR in all four analyses, there was no difference between the causal estimate before and after removing outliers (p for distortion test > 0.05) (Tables S4 and S12). The reverse directional UVMR analysis revealed that BMI, WHR, SBP and smoking heaviness had no causal effect on EA. The reason that BMI and SBP had a significant effect on EA in the IVW method might be due to horizontal pleiotropy (p for MR-Egger intercept < 0.01) (Table S5). The bias caused by sample overlap was minimal since type I error rate of four analyses were all lower than 0.06 (Table S9). The causal association between EA and four mediators were further confirmed by using a GWAS which excluded UK biobank cohort (Table S10).

### There is a harmful effect of BMI, and smoking heaviness on renal failure

In the UVMR analysis, each chosen mediator has shown a significant causal effect on KD. Each SD increase of genetically predicted BMI (OR 1.42; 95% CI 1.24, 1.64), WHR (OR 1.54; 95% CI 1.06, 2.24); SBP (OR 1.30; 95% CI 1.08, 1.56), smoking heaviness (OR 1.28; 95% CI 1.11, 1.48) was associated with a greater risk of KD. The associations were validated by other MR methods (Tables S6 and S13). The F-statistics for BMI and smoking heaviness were greater than 10, whereas those for WHR and SBP were initially less than 10 but exceeded 10 after removing outlying SNPs (Table S13). Additionally, significant heterogeneity in the IVs was observed in the BMI and SBP analyses C. No pleiotropic outliers were detected in the MR-PRESSO model, but Radial MR identified some outliers in the BMI and SBP analyses. Despite this, the causal associations between these variables and KD remained significant after removing the outliers (Table S13). Furthermore, no pleiotropy was observed in the MR-Egger analysis (Table S3).

In the MVMR model, the effect of BMI (OR 1.34; 95% CI 1.15, 1.57) and smoking heaviness (OR 1.23; 95% CI 1.07, 1.47) was slightly attenuated after adjusting for EA, while SBP (OR 1.31; 95% CI 1.08, 1.59) remained constant compared to the unadjusted model. While, a greater causal effect was observed in WHR (OR 1.77; 95% CI 1.27, 2.47) after the adjustment for EA. The MR-Egger’s result in the sensitivity analysis confirmed the association. There was no evidence of pleiotropy in all four MVMR analyses, while heterogeneity only remained significant in the analysis of BMI (Table S7). However, the causal associations between WHR and KD, as well as between SBP and KD, were no longer significant after adjusting for an alternative EA GWAS. As a result, both were excluded from the subsequent mediating proportion analysis (Table S11).

### BMI and smoking heaviness mediated the protective effect of EA on KD

The proportion of the mediation effect of each individual risk factor is shown in Figure 3. Among the two selected mediators, BMI accounted for the largest proportion, explaining 21.8% (95% CI: 9.6%, 34.0%) of the total effect of EA on KD. Smoking heaviness followed, explaining 18.7% (95% CI: 5.5%, 32.0%) of the total effect. Although significant mediation effects were observed for each individual mediator, there was no evidence that the combination of two risk factors mediated a larger proportion of the total effect (Table S8).

## Discussion

In this study, we took advantage of MR analysis method and summary-level GWAS data of large populations to evaluate the causal association between educational attainment and renal failure. We showed evidence that education played a protective role in renal failure and identified 2 causal mediators (BMI and smoking) from 24 risk factors, through which education exerted the protective effect. The study supports the notion that education may reduce the risk of renal failure by reducing BMI and smoking heaviness.

Education is a dependable indicator of socioeconomic status [9], and educational attainment is an adjustable and affective element that influences the individual’s economic position, ability to access social resources, and development of healthy lifestyles throughout their lifetime [41]. It is challenging to explore the causal effect between EA and KD, since KD is typically developed long after the completion of schooling and there are many confounding factors throughout the development of KD. However, as educational level is a genetically traceable trait, it has been proposed that MR can reveal the causal relationships between educational level and complicated diseases [42], in which method is less susceptible to confounders that are common in traditional observational studies.

Our study indicated that each increase (4.2 years) of genetically predicted educational attainment reduced approximately 32% risk of renal failure, which was generally consistent with the previous findings obtained from observational studies showing that educational attainment was negatively associated with CKD [10], and lower educational attainment could increase the incidence of CKD, the risk of ESRD as well as lower eGFR and adverse health outcomes in CKD individuals [8,43–46]. Recent MR analyses also showed similar results that educational attainment displayed a negative effect on CKD [17]. Therefore, our findings revealed that developing public policies and reducing educational inequality should be taken into account to reduce the incidence of KD and alleviate the burden of KD -associated diseases.

We identified two mediators from 24 risk factors for KD and its related diseases based on published literature. Ranked by the proportion of mediation, BMI accounted for the largest share (21.8%), followed by smoking heaviness (18.7%). These findings were generally consistent with a recent observational study, which considered WHR, BMI, hypertension, and smoking as potential mediators of the association between educational attainment and CKD [10]. Allele score–based MR was also used to find similar possible mediators in another MR study [17]. Identifying these mediators not only helps to clarify the mechanism of educational attainment’s protective effect on KD but also provides fresh insight toward clinically reducing the risk of KD. Some of these mediators are conventional risk factors, presenting practical targets that can be modified and attained, unlike EA, which is already completed when most individuals encounter the risk of renal failure. The proliferation and hypertrophy of mesangial cells as well as sensitizing the kidney to ischemic insults due to smoking [47,48] and inflammation and endothelial dysfunction because of obesity and hypertension [49] may partially explain their mediation effect in the pathway to KD. Our results of mediation analysis suggest that policies in public health to reduce obesity, and smoking might have widespread benefits on the incidence of KD which causes a huge social and economic burden.

### Limitations of the study

There are some limitations in our study: Firstly, The GWAS data utilized in this study is based on individuals of European ancestry, meaning that the findings should be cautiously applied to populations of other ethnic backgrounds. The effect of EA on KD may differ across ethnic groups, as the risk of developing kidney diseases varies by race. For instance, one study found that blacks with eGFRcreat >60 mL/min per 1.73 m^2^ at baseline exhibited a more rapid decline in kidney function than whites, even after adjusting for various potential confounders [50]. This observation highlights the need to consider ethnic differences when assessing kidney disease risk. Genetic differences between populations can result in distinct responses to environmental and genetic factors that influence KD. For example, pathogenic mutations in genes such as APOL1 and UMOD, which are associated with the onset of CKD, occur at varying frequencies across different ethnic groups, contributing to the disparities in kidney disease risk [51,52]. As such, the MR results in this study are specifically relevant to European ancestry populations, and their applicability to other populations should be carefully considered.

Secondly, although we identified two mediators and attempted to estimate their combined effect, a substantial portion (78%) of the protective association remained unexplained. This suggests that additional factors contribute to the relationship between EA and KD risk. Some plausible mediators, such as healthcare access and area poverty, were not assessed in this study, as they are not heritable traits and thus unsuitable for MR analysis [53]. Other potential mediators, such as alcohol consumption and physical inactivity—both known risk factors for kidney disease—were excluded during the screening process. However, this does not necessarily mean they do not mediate the protective effect of education on KD. Their exclusion may instead be attributed to limitations in existing GWAS data, such as insufficient sample sizes or imprecise measurements. For instance, alcohol intake frequency, only accounts for how often an individual drinks but does not capture the quantity consumed per occasion. This crude measurement may partly explain why alcohol consumption was not identified as a mediator in our analysis. Thus, higher education may exert its protective effect on KD through additional pathways that remain unaccounted for. Future research should aim to refine the measurement of potential mediators and explore alternative mechanisms to further elucidate the protective role of education in kidney health

Finally, although we used a validation GWAS in the sensitivity analysis to confirm the accuracy of the causal associations between EA and mediators, as well as between mediators and KD, potential sample overlap between GWAS studies could still impact MR estimates. In such cases, the MR results may become more similar to those from observational studies, which could undermine the reliability of causal inference in the main analysis [39]. To assess this potential bias, we calculated the proportion of sample overlap and the type I error rate. Our calculations showed that the bias was minimal and did not significantly affect the results, indicating that sample overlap had no substantial impact on our findings.

## Conclusions

In conclusion, this MR study provided evidence that there was a causal protective effect of educational attainment on renal failure, and two modifiable risk factors BMI, and SBP were identified as mediators that mediate the protective effect. These results provide the basis for interventions on these risk factors at a population level to reduce the KD risk.

## Supplementary Material

SUP_FIGURE_1.tif

Supplemental tables_20250214.docx

## Data Availability

The summary statistics can be obtained directly from each consortium (further information can be found in Materials and Methods) or accessed through the MR-Base platform (https://gwas.mrcieu.ac.uk/).

## References

[1] Levey AS, Levin A, Kellum JA. Definition and classification of kidney diseases. Am J Kidney Dis. 2013;61(5):686–688. doi:10.1053/j.ajkd.2013.03.003. 23582249

[2] Levey AS, Eckardt K-U, Tsukamoto Y, et al. Definition and classification of chronic kidney disease: a position statement from kidney disease: improving Global Outcomes (KDIGO). Kidney Int. 2005;67(6):2089–2100. doi:10.1111/j.1523-1755.2005.00365.x. 15882252

[3] Jager KJ, Kovesdy C, Langham R, et al. A single number for advocacy and communication-worldwide more than 850 million individuals have kidney diseases. Nephrol Dial Transplant. 2019;34(11):1803–1805. doi:10.1093/ndt/gfz174. 31566230

[4] Collaboration GBDCKD. Global, regional, and national burden of chronic kidney disease, 1990-2017: a systematic analysis for the Global Burden of Disease Study 2017. Lancet. 2020;395(10225):709–733. 32061315 10.1016/S0140-6736(20)30045-3 PMC7049905

[5] Susantitaphong P, Cruz DN, Cerda J, et al. World incidence of AKI: a meta-analysis. Clin J Am Soc Nephrol. 2013;8(9):1482–1493. doi:10.2215/CJN.00710113. 23744003 PMC3805065

[6] Vart P, Gansevoort RT, Joosten MM, et al. Socioeconomic disparities in chronic kidney disease: a systematic review and meta-analysis. Am J Prev Med. 2015;48(5):580–592. doi:10.1016/j.amepre.2014.11.004. 25891058

[7] Hounkpatin HO, Fraser SDS, Johnson MJ, et al. The association of socioeconomic status with incidence and outcomes of acute kidney injury. Clin Kidney J. 2020;13(2):245–252. doi:10.1093/ckj/sfz113. 32297881 PMC7147309

[8] Zeng X, Liu J, Tao S, et al. Associations between socioeconomic status and chronic kidney disease: a meta-analysis. J Epidemiol Community Health. 2018;72(4):270–279. doi:10.1136/jech-2017-209815. 29437863

[9] Galobardes B, Shaw M, Lawlor DA, et al. Indicators of socioeconomic position (part 2). J Epidemiol Community Health. 2006;60(2):95–101. doi:10.1136/jech.2004.028092. 16415256 PMC2566160

[10] Thio CHL, Vart P, Kieneker LM, et al. Educational level and risk of chronic kidney disease: longitudinal data from the PREVEND study. Nephrol Dial Transplant. 2020;35(7):1211–1218. doi:10.1093/ndt/gfy361. 30541108

[11] Saeed M, Stene LC, Reisæter AV, et al. End-stage renal disease: incidence and prediction by coronary heart disease, and educational level. Follow-up from diagnosis of childhood-onset type 1 diabetes throughout Norway 1973-2017. Ann Epidemiol. 2022;76:181–187. doi:10.1016/j.annepidem.2022.03.015. 35398254

[12] Slåtsve KB, Claudi T, Lappegård KT, et al. Level of education is associated with coronary heart disease and chronic kidney disease in individuals with type 2 diabetes: a population-based study. BMJ Open Diabetes Res Care. 2022;10(5):e002867. doi:10.1136/bmjdrc-2022-002867. PMC9528574 36171015

[13] Emdin CA, Khera AV, Kathiresan S. Mendelian randomization. JAMA. 2017;318(19):1925–1926. doi:10.1001/jama.2017.17219. 29164242

[14] Zhang Y-B, Sheng L-T, Wei W, et al. Association of blood lipid profile with incident chronic kidney disease: a Mendelian randomization study. Atherosclerosis. 2020;300:19–25. doi:10.1016/j.atherosclerosis.2020.03.020. 32276134

[15] Ye C, Kong L, Zhao Z, et al. Causal associations of obesity with chronic kidney disease and arterial stiffness: a mendelian randomization study. J Clin Endocrinol Metab. 2022;107(2):e825–e35. doi:10.1210/clinem/dgab633. 34448477

[16] Yu Z, Coresh J, Qi G, et al. A bidirectional Mendelian randomization study supports causal effects of kidney function on blood pressure. Kidney Int. 2020;98(3):708–716. doi:10.1016/j.kint.2020.04.044. 32454124 PMC7784392

[17] Park S, Lee S, Kim Y, et al. Causal effects of education on chronic kidney disease: a Mendelian randomization study. Clin Kidney J. 2021;14(8):1932–1938. doi:10.1093/ckj/sfaa240. 34345417 PMC8323131

[18] Hemani G, Bowden J, Davey Smith G. Evaluating the potential role of pleiotropy in Mendelian randomization studies. Hum Mol Genet. 2018;27(R2):R195–R208. doi:10.1093/hmg/ddy163. 29771313 PMC6061876

[19] Zhang J, Chen Z, Pärna K, et al. Mediators of the association between educational attainment and type 2 diabetes mellitus: a two-step multivariable Mendelian randomisation study. Diabetologia. 2022;65(8):1364–1374. doi:10.1007/s00125-022-05705-6. 35482055 PMC9283137

[20] Lee JJ, Wedow R, Okbay A, et al. Gene discovery and polygenic prediction from a genome-wide association study of educational attainment in 1.1 million individuals. Nat Genet. 2018;50(8):1112–1121. doi:10.1038/s41588-018-0147-3. 30038396 PMC6393768

[21] Kurki MI, Karjalainen J, Palta P, et al. FinnGen provides genetic insights from a well-phenotyped isolated population. Nature. 2023;613(7944):508–518. doi:10.1038/s41586-022-05473-8. 36653562 PMC9849126

[22] Yengo L, Sidorenko J, Kemper KE, et al. Meta-analysis of genome-wide association studies for height and body mass index in approximately 700000 individuals of European ancestry. Hum Mol Genet. 2018;27(20):3641–3649. doi:10.1093/hmg/ddy271. 30124842 PMC6488973

[23] Shungin D, Winkler TW, Croteau-Chonka DC, et al. New genetic loci link adipose and insulin biology to body fat distribution. Nature. 2015;518(7538):187–196. doi:10.1038/nature14132. 25673412 PMC4338562

[24] Scott RA, Lagou V, Welch RP, et al. Large-scale association analyses identify new loci influencing glycemic traits and provide insight into the underlying biological pathways. Nat Genet. 2012;44(9):991–1005. doi:10.1038/ng.2385. 22885924 PMC3433394

[25] Liu M, Jiang Y, Wedow R, et al. Association studies of up to 1.2 million individuals yield new insights into the genetic etiology of tobacco and alcohol use. Nat Genet. 2019;51(2):237–244. doi:10.1038/s41588-018-0307-5. 30643251 PMC6358542

[26] Richardson TG, Sanderson E, Palmer TM, et al. Evaluating the relationship between circulating lipoprotein lipids and apolipoproteins with risk of coronary heart disease: a multivariable Mendelian randomisation analysis. PLoS Med. 2020;17(3):e1003062. doi:10.1371/journal.pmed.1003062. 32203549 PMC7089422

[27] Klimentidis YC, Raichlen DA, Bea J, et al. Genome-wide association study of habitual physical activity in over 377,000 UK Biobank participants identifies multiple variants including CADM2 and APOE. Int J Obes (Lond). 2018;42(6):1161–1176. doi:10.1038/s41366-018-0120-3. 29899525 PMC6195860

[28] Hemani G, Zheng J, Elsworth B, et al. The MR-Base platform supports systematic causal inference across the human phenome. Elife. 2018;7. doi:10.7554/eLife.34408. PMC5976434 29846171

[29] Qing J, Li Y, Soliman KM, et al. A practical guide for nephrologist peer reviewers: understanding and appraising Mendelian randomization studies. Ren Fail. 2025;47(1):2445763. 39806780 10.1080/0886022X.2024.2445763 PMC11734392

[30] Burgess S, Dudbridge F, Thompson SG. Combining information on multiple instrumental variables in Mendelian randomization: comparison of allele score and summarized data methods. Stat Med. 2016;35(11):1880–1906. doi:10.1002/sim.6835. 26661904 PMC4832315

[31] Pierce BL, Ahsan H, Vanderweele TJ. Power and instrument strength requirements for Mendelian randomization studies using multiple genetic variants. Int J Epidemiol. 2011;40(3):740–752. doi:10.1093/ije/dyq151. 20813862 PMC3147064

[32] Burgess S, Thompson SG, CRP CHD Genetics Collaboration. Avoiding bias from weak instruments in Mendelian randomization studies. Int J Epidemiol. 2011;40(3):755–764. doi:10.1093/ije/dyr036. 21414999

[33] Relton CL, Davey Smith G. Two-step epigenetic Mendelian randomization: a strategy for establishing the causal role of epigenetic processes in pathways to disease. Int J Epidemiol. 2012;41(1):161–176. doi:10.1093/ije/dyr233. 22422451 PMC3304531

[34] MacKinnon DP, Fairchild AJ, Fritz MS. Mediation analysis. Annu Rev Psychol. 2007;58(1):593–614. doi:10.1146/annurev.psych.58.110405.085542. 16968208 PMC2819368

[35] Burgess S, Thompson SG. Interpreting findings from Mendelian randomization using the MR-Egger method. Eur J Epidemiol. 2017;32(5):377–389. doi:10.1007/s10654-017-0255-x. 28527048 PMC5506233

[36] Bowden J, Davey Smith G, Haycock PC, et al. Consistent estimation in Mendelian randomization with some invalid instruments using a weighted median estimator. Genet Epidemiol. 2016;40(4):304–314. doi:10.1002/gepi.21965. 27061298 PMC4849733

[37] Verbanck M, Chen CY, Neale B, et al. Detection of widespread horizontal pleiotropy in causal relationships inferred from Mendelian randomization between complex traits and diseases. Nat Genet. 2018;50(5):693–698. doi:10.1038/s41588-018-0099-7. 29686387 PMC6083837

[38] Okbay A, Beauchamp JP, Fontana MA, et al. Genome-wide association study identifies 74 loci associated with educational attainment. Nature. 2016;533(7604):539–542. doi:10.1038/nature17671. 27225129 PMC4883595

[39] Burgess S, Davies NM, Thompson SG. Bias due to participant overlap in two-sample Mendelian randomization. Genet Epidemiol. 2016;40(7):597–608. doi:10.1002/gepi.21998. 27625185 PMC5082560

[40] Savage JE, Jansen PR, Stringer S, et al. Genome-wide association meta-analysis in 269,867 individuals identifies new genetic and functional links to intelligence. Nat Genet. 2018;50(7):912–919. doi:10.1038/s41588-018-0152-6. 29942086 PMC6411041

[41] Lawrence EM. Why do college graduates behave more healthfully than those who are less educated? J Health Soc Behav. 2017;58(3):291–306. doi:10.1177/0022146517715671. 28845056 PMC5570614

[42] Tillmann T, Vaucher J, Okbay A, et al. Education and coronary heart disease: mendelian randomisation study. BMJ. 2017;358:j3542. doi:10.1136/bmj.j3542. 28855160 PMC5594424

[43] Morton RL, Schlackow I, Staplin N, et al. Impact of educational attainment on health outcomes in moderate to severe CKD. Am J Kidney Dis. 2016;67(1):31–39. doi:10.1053/j.ajkd.2015.07.021. 26385817 PMC4685934

[44] Hsu CY, Iribarren C, McCulloch CE, et al. Risk factors for end-stage renal disease: 25-year follow-up. Arch Intern Med. 2009;169(4):342–350. doi:10.1001/archinternmed.2008.605. 19237717 PMC2727643

[45] Lash JP, Go AS, Appel LJ, et al. Chronic Renal Insufficiency Cohort (CRIC) study: baseline characteristics and associations with kidney function. Clin J Am Soc Nephrol. 2009;4(8):1302–1311. doi:10.2215/CJN.00070109. 19541818 PMC2723966

[46] Adjei DN, Stronks K, Adu D, et al. Relationship between educational and occupational levels, and chronic kidney disease in a multi-ethnic sample- The HELIUS study. PLoS ONE. 2017;12(11):e0186460. doi:10.1371/journal.pone.0186460. 29091928 PMC5665422

[47] Arany I, Grifoni S, Clark JS, et al. Chronic nicotine exposure exacerbates acute renal ischemic injury. Am J Physiol Renal Physiol. 2011;301(1):F125–F133. doi:10.1152/ajprenal.00041.2011. 21511693 PMC3129886

[48] Rezonzew G, Chumley P, Feng W, et al. Nicotine exposure and the progression of chronic kidney disease: role of the alpha7-nicotinic acetylcholine receptor. Am J Physiol Renal Physiol. 2012;303(2):F304–12. doi:10.1152/ajprenal.00661.2011. 22552933 PMC3404588

[49] Redon J, Lurbe E. The kidney in obesity. Curr Hypertens Rep. 2015;17(6):555. doi:10.1007/s11906-015-0555-z. 25893477

[50] Peralta CA, Katz R, DeBoer I, et al. Racial and ethnic differences in kidney function decline among persons without chronic kidney disease. J Am Soc Nephrol. 2011;22(7):1327–1334. doi:10.1681/ASN.2010090960. 21700831 PMC3137580

[51] Devuyst O, Pattaro C. The UMOD locus: insights into the pathogenesis and prognosis of kidney disease. J Am Soc Nephrol. 2018;29(3):713–726. doi:10.1681/ASN.2017070716. 29180396 PMC5827601

[52] Umeukeje EM, Young BA. Genetics and ESKD disparities in African Americans. Am J Kidney Dis. 2019;74(6):811–821. doi:10.1053/j.ajkd.2019.06.006. 31606237 PMC7373097

[53] Zajacova A, Lawrence EM. The relationship between education and health: reducing disparities through a contextual approach. Annu Rev Public Health. 2018;39(1):273–289. doi:10.1146/annurev-publhealth-031816-044628. 29328865 PMC5880718

