# Supplementary figures and images for: Educational attainment, body mass index, and smoking as mediators in kidney disease risk: a two-step Mendelian randomization study
Source: Ren Fail. 2025 Mar 11;47(1):2476051. doi: 10.1080/0886022X.2025.2476051 (PMC11899219; doi:10.1080/0886022X.2025.2476051)

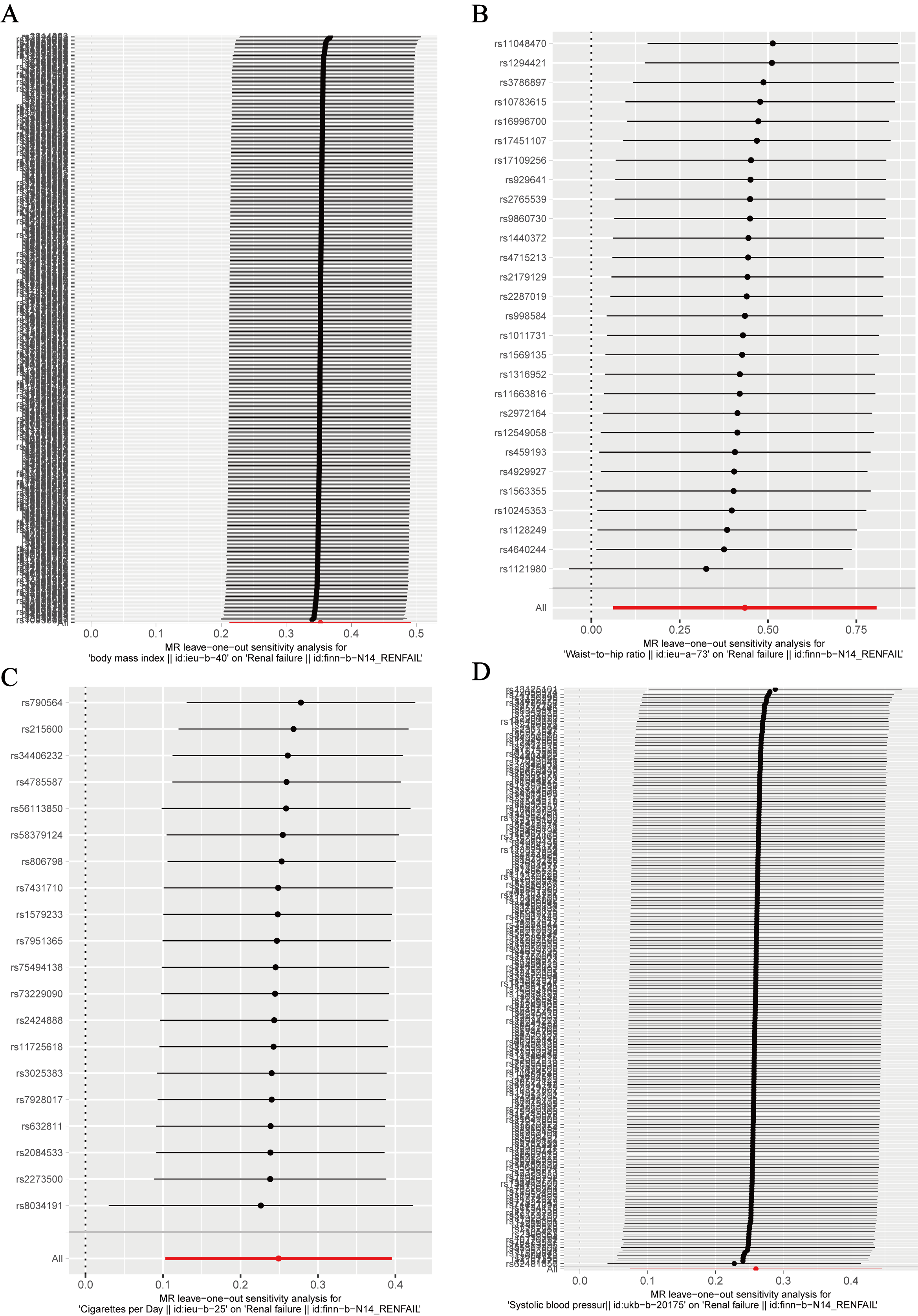

Supplement: SUP_FIGURE_1.tif [file IRNF_A_2476051_SM9813.tif]
