# Supplementary material for: Educational attainment, body mass index, and smoking as mediators in kidney disease risk: a two-step Mendelian randomization study
Source: Ren Fail. 2025 Mar 11;47(1):2476051. doi: 10.1080/0886022X.2025.2476051 (PMC11899219; doi:10.1080/0886022X.2025.2476051)
Supplement: Supplemental tables_20250214.docx [file IRNF_A_2476051_SM9812.docx]

**Supplemental Table 1.** Epidemiological evidence of the association between 20 candidate mediators and Kidney disease or its subitems.

| **Mediators** | **Epidemiological evidence** | **PMID** |
| --- | --- | --- |
| Body mass index | In a cohort of Chinese men and women in Singapore, the risk of developing ESRD was assessed based on BMI categories. The HR(95%CI) for those with BMIs under 18.5, 23 to under 27.5, and 27.5 kg/m2 or more were 0.54 (0.37, 0.79), 1.40 (1.20, 1.64), and 2.13 (1.74, 2.59), respectively, compared to those with a normal BMI of 18.5 to under 23 kg/m2. | 28528130 |
| Waist-to-hip ratio | A recent study conducted by the Atherosclerosis Risk in Communities (ARIC) found that after adjustment, higher waist-to-hip ratios were associated with steeper declines in estimated glomerular filtration rate (eGFR) in both Black men and women, with differences of -1.60 (95% CI, -2.42 to -0.78) and -1.50 (95% CI, -2.05 to -0.95), respectively. Meanwhile, White women showed a smaller difference of -0.82 (95% CI, -1.06 to -0.58), and White men showed no significant difference at -0.25 (95% CI, -0.50 to 0.01). | 32979415 |
| Hypertension | In a large cohort of 2,144,801 participants, the risk of ESRD increased with the burden of hypertension. After adjustment, compared to those with a hypertension burden of 0, individuals with hypertension burden of 1, 2, 3, and 4 had adjusted hazard ratios (HRs) of 1.35, 1.54, 1.51, and 2.28, respectively, for the development of ESRD | 34408283 |
| Systolic blood pressure | Significant ESRD risk was associated with 3-year SBP 130 mmHg (2.37 [1.23-4.56]). In an Okinawa community-based cohort, those with baseline SBP 130 mmHg and 1- to 3-year SBP 140 mmHg had a substantial risk of developing end-stage renal disease. | 31385160 |
| Diastolic blood pressure | The incidence rates of ESRD rose with rising SBP and DBP during a median follow-up of 7.15 years. In Korea, diabetic patients under 40 with a DBP of 100 mm Hg had the greatest HR for ESRD. | 35607989 |
| Smoking initiation | After adjusting for confounding variables, the HR (95% CI) for the development of CKD in former and current smokers, respectively, were 1.13 (0.95-1.35) and 1.26 (1.07-1.48), compared to never-smokers, from the Korean Genome and Epidemiology Study. | 32853266 |
| Smoking heaviness | Smoking was identified as an independent risk factor for poor renal development in IgAN (HR = 1.58; p = 0.043) by multivariate Cox regression analysis from West China Hospital. | 34187402 |
| HDL-C | Low HDL-C was significantly associated with a≥ 30% eGFR decrease or ESRD in CKD patients, according to multivariate Cox regression analysis (HR= 4.80, P = 0.009). | 30874893 |
| LDL-C | A Chinese retrospective study found that elevated LDL levels were associated with an increased risk of ESRD progression in both general chronic kidney disease (CKD) patients (HR=2.494, P<0.001) and CKD patients without diabetes (HR=3.982, P<0.001). | 36046149 |
| Triglycerides | Persons with diabetes had a stronger relationship between higher TG:HDL-C ratio and deterioration in eGFR (P for interaction = 0.002) and incidence CKD (P for interaction = 0.05) than participants without diabetes from a longitudinal cohort study in Japan. | 26145254 |
| High cholesterol | In CKD patients, TC/HDL-C was independently linked with the progression of the disease (OR 1.345; 95% CI 1.079-1.677) from a retrospective, case-control study in China. | 35098433 |
| Urate | In a retrospective cohort research, gout was linked to a higher risk of developing incident CKD (adjusted HR 1.78 95% CI 1.70 to 1.85) | 30376864 |
| Diabetes | For HD, the adjusted HR of incident DM was 0.46 (95% CI 0.37-0.58, p = 0.0001), and for PD, it was 0.84 (95% CI 0.47-1.51, p = 0.56) from a Taiwanese study. | 30314341 |
| Fasting glucose | The IRR for CKD for IGT/IFG compared to normoglycaemia was 4.0 (95% CI, 3.2 to 5.1) in a cohort of 40092 IGT/IFG patients, | 29482513 |
| Alcohol consumption | In IgA nephropathy patients, a history of alcohol consumption (within 1 year) was found to be independently associated with an increased risk of ESKD, with an OR of 1.32 (p<0.05), according to a recent study. Conversely, physical activity habits were linked to a lower risk of ESKD, with a multivariate logistic analysis showing an OR of 0.06 (p<0.05). | 31580172 |
| Physical activities |  |  |
| TV watching | The Health, Aging, and Body Composition study found that individuals who reported watching television for more than three hours per day had a higher risk of incident CKD compared to those who watched television for less than two hours per day, with an adjusted hazard ratio (HR) of 1.34 (95% CI, 1.09-1.65). | 24762526 |
| Computer using | Longer SB was independently related with a greater OR of CKD after adjustment (adjusted OR of SB>16 h/day: 1.81 [95%CI: 1.52-2.15] compared to SB<7 h/day) in a Japanese cross-sectional study. | 34657911 |
| Sleep duration | Shorter sleep duration (≤5 hours) and longer sleep length (≥8 hours) were both related with ESKD. The adjusted HR (95% CI) for sleep durations of 5.0 and 8.0 hours was 2.05 (1.31, 3.21) and 1.48 (1.01, 2.16) respectively from a 4-year prospective cohort study. | 30442866 |
| Urinary potassium | In a cohort of 855 CKD patients, the adjusted HR for 24-hour urine potassium excretion were 3.05 (95% confidence interval, 1.54 to 6.04) when comparing the lowest quartile with the highest quartile. | 30765533 |
| Dietary pattern | Seventeen studies, with a total of 149,958 participants, were included in the systematic review and meta-analysis. The results showed that those in the highest category of a healthy dietary pattern had a significantly lower risk of CKD compared to those in the lowest category (OR = 0.69; CI: 0.57, 0.84). | 33419440 |

HR, hazard ratio; ESRD, end-stage renal disease; eGFR, estimated glomerular filtration rate; SD, standard deviation; HDL-C, high-density lipoprotein cholesterol; CI, confidence interval; SBP, systolic blood pressure; DBP, diastolic blood pressure ; CKD, chronic kidney disease; IgAN, IgA nephropathy; LDL, high-density lipoprotein; TG, triglycerides; TC, total cholesterol; DM, diabetes mellitus; HD, hemodialysis; IRR, incidence rate ratio; PD, peritoneal dialysis; IGT, impaired glucose tolerance; ESKD, end-stage Kidney disease; IFG, impaired fasting glucose; OR, odd ratio; SB, sedentary behavior.

**Supplemental Table 2.** Heterogeneity test of all UVMR analyses in the study.

| **Exposures** | **Outcomes** | **Method** | **Q** | **Q_df** | **Q_p-value** |
| --- | --- | --- | --- | --- | --- |
| Educational attainment | Kidney disease | MR Egger | 245.885 | 255 | 6.48E-01 |
|  |  | Inverse variance weighted | 246.886 | 256 | 6.47E-01 |
|  | Waist-to-hip ratio | MR Egger | 295.191 | 210 | 9.55E-05 |
|  |  | Inverse variance weighted | 295.301 | 211 | 1.13E-04 |
|  | Body mass index | MR Egger | 2071.008 | 204 | 7.70E-306 |
|  |  | Inverse variance weighted | 2072.737 | 205 | 1.13E-305 |
|  | Smoking heaviness | MR Egger | 552.861 | 254 | 2.98E-24 |
|  |  | Inverse variance weighted | 555.356 | 255 | 2.23E-24 |
|  | Systolic blood pressure | MR Egger | 809.874 | 255 | 5.10E-59 |
|  |  | Inverse variance weighted | 812.336 | 256 | 3.91E-59 |
| Waist-to-hip ratio | Kidney disease | MR Egger | 37.537 | 26 | 6.68E-02 |
|  |  | Inverse variance weighted | 38.655 | 27 | 6.81E-02 |
| Body mass index |  | MR Egger | 524.836 | 426 | 7.43E-04 |
|  |  | Inverse variance weighted | 525.199 | 427 | 8.03E-04 |
| Smoking heaviness |  | MR Egger | 18.056 | 18 | 4.52E-01 |
|  |  | Inverse variance weighted | 18.081 | 19 | 5.17E-01 |
| Systolic blood pressure |  | MR Egger | 273.026 | 214 | 3.93E-03 |
|  |  | Inverse variance weighted | 274.117 | 215 | 3.95E-03 |

MR, Mendelian randomization

**Supplemental Table 3.** Directional pleiotropy test and statistic power of main UVMR analyses in the study.

| **Exposures** | **Outcomes** | **MR-Egger_intercept** | **Se** | **P value** | **Statistic power** |
| --- | --- | --- | --- | --- | --- |
| Educational attainment | Kidney disease | 0.005 | 0.005 | 0.318 | 97.5% |
|  | Waist-to-hip ratio | 0.000 | 0.002 | 0.780 | 100% |
|  | Body mass index | 0.001 | 0.002 | 0.680 | 100% |
|  | Smoking heaviness | -0.002 | 0.002 | 0.285 | 100% |
|  | Systolic blood pressure | -0.001 | 0.001 | 0.380 | 100% |
| Waist-to-hip ratio | Kidney disease | -0.019 | 0.022 | 0.387 | 78.3% |
| Body mass index |  | -0.002 | 0.003 | 0.588 | 100% |
| Smoking heaviness |  | 0.001 | 0.009 | 0.877 | 92.9% |
| Systolic blood pressure |  | -0.005 | 0.005 | 0.356 | 88.9% |

MR, Mendelian randomization

**Supplemental Table 4.** MR-PRESSO’s partial result of all UVMR analyses in the study.

| **Exposures** | **Outcomes** | **Number of outliers** | **P value for distortion test** |
| --- | --- | --- | --- |
| Educational attainment | Kidney disease | NA | NA |
|  | Waist-to-hip ratio | 1 | 0.603 |
|  | Body mass index | 38 | 0.114 |
|  | Smoking heaviness | 4 | 0.624 |
|  | Systolic blood pressure | 9 | 0.455 |
|  | High cholesterol | 2 | 0.6385 |
|  | Triglycerides | 17 | 0.611625 |
|  | urate | 10 | 0.84625 |
|  | Alcohol drinking | 11 | 0.365625 |
|  | Sleeping duration | 21 | 0.6008333 |
|  | MVPA | 12 | 0.41275 |
|  | HDL-C | 19 | 0.932 |
|  | Fasting glucose | 7 | 0.10525 |
|  | Salt intake | 16 | 0.885 |
|  | Processed food intake | 6 | 0.894 |
| Waist-to-hip ratio | Kidney disease | NA | NA |
| Body mass index |  | NA | NA |
| Smoking heaviness |  | NA | NA |
| Systolic blood pressure |  | NA | NA |
| High cholesterol |  | NA | 0.314 |
| Triglycerides |  | NA | 0.13925 |
| urate |  | NA | NA |
| Alcohol drinking |  | NA | NA |
| Sleeping duration |  | NA | NA |
| MVPA |  | NA | NA |
| HDL-C |  | NA | NA |
| Fasting glucose |  | NA | NA |
| Salt intake |  | NA | NA |
| Processed food intake |  | NA | NA |

**Supplemental Table 5.** Reverse MR analyses of the effect of each mediator on educational attainment.

| **Exposures** | **Outcome** | **Methods** | **β(95%CI)** | **P value** | **MR-Egger intercept** | **P value for MR-Egger intercept** |
| --- | --- | --- | --- | --- | --- | --- |
| Waist-to-hip ratio | Educational attainment | MR Egger | 0.263(0.075,0.451) | 0.011 | -0.007 | 0.008 |
|  |  | Weighted median | 0.019(-0.029,0.067) | 0.442 |  |  |
|  |  | Inverse variance weighted | -0.006(-0.052,0.040) | 0.786 |  |  |
| Body mass index |  | MR Egger | -0.039(-0.112,0.034) | 0.293 | -0.002 | 0.004 |
|  |  | Weighted median | -0.096(-0.121,-0.072) | 0.000 |  |  |
|  |  | Inverse variance weighted | -0.138(-0.166,-0.109) | 0.000 |  |  |
| Smoking heaviness |  | MR Egger | 0.058(0.011,0.105) | 0.027 | -0.008 | 0.000 |
|  |  | Weighted median | -0.001(-0.017,0.016) | 0.930 |  |  |
|  |  | Inverse variance weighted | -0.038(-0.079,0.002) | 0.064 |  |  |
| Systolic blood pressure |  | MR Egger | 0.031(-0.063,0.125) | 0.518 | -0.002 | 0.022 |
|  |  | Weighted median | -0.047(-0.074,-0.019) | 0.001 |  |  |
|  |  | Inverse variance weighted | -0.073(-0.105,-0.041) | 0.000 |  |  |

CI, confidence interval; MR, Mendelian randomization.

**Supplemental Table 6.** Complementary methods’ result of all UVMR analyses in the study. OR, odds ratio; CI, confidence interval; MR-PRESSO, Mendelian randomization pleiotropy residual sum and outlier; MR, Mendelian randomization.

| **Exposures** | **Outcomes** | **Methods** | **β(95%CI)** | **OR(95%CI)** | **P value** |
| --- | --- | --- | --- | --- | --- |
| Educational attainment | Waist-to-hip ratio | MR Egger | -0.199(-0.434,0.036) | \ | 9.80E-02 |
|  |  | Weighted median | -0.244(-0.317,-0.171) | \ | 5.60E-11 |
|  |  | MR-PRESSO | -0.237(-0.287,-0.187) | \ | 9.99E-18 |
|  | Body mass index | MR Egger | -0.340(-0.604,-0.076) | \ | 1.23E-02 |
|  |  | Weighted median | -0.242(-0.285,-0.200) | \ | 8.96E-29 |
|  |  | MR-PRESSO | -0.282(-0.337,-0.227) | \ | 3.79E-20 |
|  | Smoking heaviness | MR Egger | -0.170(-0.500,0.160) | \ | 3.14E-01 |
|  |  | Weighted median | -0.282(-0.375,-0.188) | \ | 3.58E-09 |
|  |  | MR-PRESSO | -0.343(-0.418,-0.268) | \ | 3.15E-17 |
|  | Systolic blood pressure | MR Egger | -0.069(-0.219,0.081) | \ | 3.71E-01 |
|  |  | Weighted median | -0.143(-0.181,-0.105) | \ | 1.14E-13 |
|  |  | MR-PRESSO | -0.134(-0.168,-0.099) | \ | 3.63E-13 |
| Waist-to-hip ratio | Kidney disease | MR Egger | 1.196(-0.509,2.847) | 3.218(0.601.17.231) | 1.84E-01 |
|  |  | Weighted median | 0.592(0.101,1.083) | 1.808(1.107,2.952) | 1.80E-02 |
|  |  | MR-PRESSO | 0.333(-0.037,0.702) | 1.395(0.964,2.018) | 8.87E-02 |
| Body mass index |  | MR Egger | 0.445(0.083,0.807) | 1.560(1.086,2,241) | 1.64E-02 |
|  |  | Weighted median | 0.431(0.205,0.656) | 1.538(1.228,1.926) | 1.78E-04 |
|  |  | MR-PRESSO | 0.350(0.482,0.218) | 1.419(1.244,1.619) | 2.80E-07 |
| Smoking heaviness |  | MR Egger | 0.233(-0.020,0.486) | 1.262(0.980,1.626) | 8.84E-02 |
|  |  | Weighted median | 0.265(0.069,0.460) | 1.303(1.072,1.584) | 7.92E-03 |
|  |  | MR-PRESSO | 0.239(0.108,0.370) | 1.270(1.114,1.448) | 1.67E-03 |
| Systolic blood pressure |  | MR Egger | 0.504(-0.046,1.053) | 1.655(0.955,2.867) | 7.39E-02 |
|  |  | Weighted median | 0.259(-0.001,0.520) | 1.296(0.999,1.682) | 5.13E-02 |
|  |  | MR-PRESSO | 0.242(0.062,0.422) | 1.274(1.064,1.526) | 9.00E-03 |

**Supplemental Table 7.** MR-Egger’s result and heterogeneity test for mediator’s effect on Kidney disease adjusted for education.

| **Exposures** | **Outcome** | **Method** | **β(95%CI)** | **P value** | **Int** | **P-int** | **Q** | **Q p-value** |
| --- | --- | --- | --- | --- | --- | --- | --- | --- |
| Waist-to-hip ratio | Kidney disease | MR Egger | 0.566(0.234,0.899) | 8.47E-04 | 0.002 | 7.18E-01 | 234.672 | 2.67E-01 |
| Body mass index |  |  | 0.300(0.144,0.455) | 1.61E-04 | 0.001 | 5.36E-01 | 509.051 | 4.64E-03 |
| Smoking heaviness |  |  | 0.218(0.074,0.361) | 2.95E-03 | 0.006 | 1.76E-01 | 252.467 | 6.36E-01 |
| Systolic blood pressure |  |  | 0.273(0.081,0.464) | 5.26E-03 | 0.001 | 7.14E-01 | 322.321 | 3.32E-01 |

CI, confidence interval; IVW, inverse variance weighted; Int, MR-Egger intercept; P-int, P value for MR-Egger intercept; Q, IVW Q statistic

**Supplemental Table 8.** Combined mediated proportion of multiple mediators.

| **Mediators** | **Mediated proportion(95%CI)** |
| --- | --- |
| BMI+smoking | 0.133(-0.669,0.936) |

CI, confidence interval; BMI, body mass index; WHR, waist-to-hip ratio; SBP, systolic blood pressure.

**Supplemental Table 9.** Type 1 error rate of analyses of EA on BMI, WHR, smoking heaviness and SBP.

| **Exposure** | **Outcomes** | **Sample size** | **Overlap proportion** | **Type I error** |
| --- | --- | --- | --- | --- |
| EA | BMI | 1447620 | 0.315 | 0.05^*^ |
|  | WHR | 978589 | 0.012 | 0.05^*^ |
|  | Smoking heaviness | 1103679 | 0.115 | 0.05^*^ |
|  | SBP | 1202764 | 0.363 | 0.05^*^ |

BMI, body mass index; WHR, waist-to-hip ratio; SBP, systolic blood pressure. * The results are displayed with only two decimal places, not rounded, as the calculation website only provides values up to two decimal places.

**Supplemental Table 10.**  UVMR results in the sensitivity analysis by using alternative educational attainment GWAS data.

| **Exposure** | **Outcomes** | **Methods** | **β(95%CI)** | **OR(95%CI)** | **P value** |
| --- | --- | --- | --- | --- | --- |
| Educational attainment | Body mass index | Inverse variance weighted | -0.301(-0.419,-0.183) | \ | 5.83E-07 |
|  | Waist-to-hip ratio | Inverse variance weighted | -0.306(-0.394,-0.218) | \ | 1.10E-11 |
|  | Smoking heaviness | Inverse variance weighted | -0.305(-0.439,-0.170) | \ | 8.65E-06 |
|  | Systolic blood pressure | Inverse variance weighted | -0.134(-0.193,-0.075) | \ | 8.83E-06 |
|  | Kidney disease | Inverse variance weighted | -0.336(-0.640,-0.033) | 0.714 (0.527,0.968) | 2.99E-02 |

**Supplemental Table 11.** MVMR results of effects of mediators on KD adjusted by alternative educational attainment GWAS data in the sensitivity analysis.

| **Outcome** | **Exposures** | **Methods** | **β(95%CI)** | **OR(95%CI)** | **P value** |
| --- | --- | --- | --- | --- | --- |
| Kidney disease | Body mass index | Inverse variance weighted | 0.343(0.189,0.498) | 1.410(1.208,1.645) | 1.27E-05 |
|  | Waist-to-hip ratio | Inverse variance weighted | 0.318(-0.002,0.638) | 1.375(0.998,1.894) | 5.11E-02 |
|  | Smoking heaviness | Inverse variance weighted | 0.198(0.065,0.330) | 1.219(1.067,1.391) | 3.46E-03 |
|  | Systolic blood pressure | Inverse variance weighted | 0.183(-0.003,0.368) | 1.201(0.997,1.445) | 5.36E-02 |

**Supplemental Table 12.** Mediated proportion of mediators in the sensitivity analysis. CI, confidence interval.

| **Mediators** | **Mediated proportion(95%CI)** |
| --- | --- |
| Body mass index | 0.307 (0.124, 0.490) |
| Waist-to-hip ratio | 0.290 (-0.013, 0.592) |
| Smoking heaviness | 0.179 (0.035, 0.323) |
| Systolic blood pressure | 0.073 (-0.008, 0.153) |

**Supplemental Table 13.** Partial results of radial MR analysis of all.

| **Exposures** | **Outcomes** | **Before outliers removal** | | | | | | **After outliers removal** | | | | | |
| --- | --- | --- | --- | --- | --- | --- | --- | --- | --- | --- | --- | --- | --- |
|  |  | Estimate | p-value | F-statistic | p-value | Q-Statistic | p-value | Estimate | p-value | F-statistic | p-value | Q-Statistic | p-value |
| EA | KD | -0.388 | 0.000 | 15.140 | 0.000 | 246.886 | 0.647 | -0.373 | 0.000 | 17.940 | 0.000 | 176.653 | 1.000 |
|  | BMI | -0.286 | 0.000 | 81.290 | <2e-16 | 2072.737 | 0.000 | -0.295 | 0.000 | 387.130 | <2e-16 | 127.772 | 0.132 |
|  | WHR | -0.231 | 0.000 | 66.000 | 0.000 | 295.301 | 0.000 | -0.250 | 0.000 | 122.740 | <2e-16 | 148.406 | 0.989 |
|  | SH | -0.344 | 0.000 | 65.780 | 0.000 | 555.356 | 0.000 | -0.335 | 0.000 | 116.190 | <2e-16 | 205.296 | 0.671 |
|  | SBP | -0.134 | 0.000 | 48.440 | 0.000 | 812.335 | 0.000 | -0.147 | 0.000 | 116.050 | <2e-16 | 227.011 | 0.052 |
| BMI | KD | 0.352 | 0.000 | 24.470 | 0.000 | 525.199 | 0.001 | 0.348 | 0.000 | 32.030 | 0.000 | 333.551 | 0.990 |
| WHR |  | 0.434 | 0.022 | 5.220 | 0.030 | 38.655 | 0.068 | 0.538 | 0.001 | 10.970 | 0.003 | 22.380 | 0.557 |
| SH |  | 0.249 | 0.001 | 11.730 | 0.003 | 18.081 | 0.517 | 0.278 | 0.000 | 24.450 | 0.000 | 10.042 | 0.931 |
| SBP |  | 0.260 | 0.006 | 7.520 | 0.007 | 274.117 | 0.004 | 0.318 | 0.000 | 16.360 | 0.000 | 150.300 | 0.992 |

**Supplemental Table 14.** SNP list used in the MR analysis of educational attainment and Kidney disease.

| a | SNP | beta.exposure | eaf.exposure | pval.outcome | pval.exposure |
| --- | --- | --- | --- | --- | --- |
| 1 | rs10073890 | -0.01262 | 0.7364 | 0.8453 | 1.11E-10 |
| 2 | rs1008078 | -0.01738 | 0.4099 | 0.1189 | 1.20E-23 |
| 3 | rs10189857 | -0.01725 | 0.4184 | 0.3572 | 6.70E-24 |
| 4 | rs10191758 | 0.01631 | 0.381 | 0.6723 | 9.60E-21 |
| 5 | rs10205801 | -0.01053 | 0.5068 | 0.2275 | 7.17E-10 |
| 6 | rs10215082 | 0.01303 | 0.5612 | 0.9575 | 3.33E-14 |
| 7 | rs10240905 | 0.01167 | 0.6684 | 0.606001 | 3.79E-11 |
| 8 | rs10456918 | 0.01485 | 0.182 | 0.7391 | 3.67E-11 |
| 9 | rs10460095 | -0.01066 | 0.5867 | 0.1867 | 4.87E-10 |
| 10 | rs1051474 | 0.01301 | 0.2738 | 0.095041 | 4.86E-12 |
| 12 | rs10765775 | 0.01488 | 0.3963 | 0.991 | 2.62E-17 |
| 15 | rs10798418 | -0.00957 | 0.466 | 0.770999 | 3.29E-08 |
| 16 | rs10856785 | -0.01132 | 0.7296 | 0.2133 | 3.83E-09 |
| 19 | rs10887801 | 0.01087 | 0.4371 | 0.8066 | 2.27E-10 |
| 20 | rs10940921 | -0.01089 | 0.5697 | 0.2291 | 7.00E-10 |
| 22 | rs10994777 | 0.0146 | 0.1395 | 0.7618 | 3.36E-10 |
| 23 | rs11023749 | 0.01132 | 0.6701 | 0.3945 | 2.96E-10 |
| 24 | rs1105307 | -0.01173 | 0.2449 | 0.3797 | 1.67E-09 |
| 25 | rs1106090 | 0.01173 | 0.6259 | 0.43 | 2.09E-11 |
| 26 | rs11081529 | -0.01311 | 0.2568 | 0.2026 | 1.82E-12 |
| 27 | rs11123818 | 0.02081 | 0.3946 | 0.8234 | 1.72E-32 |
| 28 | rs111821073 | 0.01385 | 0.1633 | 0.1616 | 4.85E-09 |
| 29 | rs112687095 | 0.01325 | 0.165 | 0.4826 | 2.42E-08 |
| 31 | rs113182709 | 0.03225 | 0.02381 | 0.5757 | 1.29E-08 |
| 32 | rs113520408 | 0.01304 | 0.2857 | 0.9669 | 1.02E-11 |
| 33 | rs113615161 | -0.01472 | 0.1395 | 0.1239 | 3.97E-09 |
| 34 | rs1143770 | 0.01136 | 0.5918 | 0.1101 | 4.31E-11 |
| 36 | rs115454970 | -0.01185 | 0.3044 | 0.2285 | 2.76E-09 |
| 37 | rs11601122 | -0.01947 | 0.1497 | 0.1136 | 2.24E-17 |
| 38 | rs11620355 | 0.01756 | 0.1156 | 0.2758 | 4.77E-09 |
| 39 | rs11627087 | -0.01788 | 0.08503 | 0.3663 | 3.71E-08 |
| 40 | rs11635092 | -0.01231 | 0.3639 | 0.4425 | 3.89E-12 |
| 41 | rs11657342 | 0.01404 | 0.3554 | 0.8538 | 1.94E-13 |
| 42 | rs11663602 | -0.01213 | 0.2568 | 0.8633 | 1.64E-10 |
| 43 | rs11678980 | -0.01744 | 0.4456 | 0.097931 | 4.29E-24 |
| 44 | rs11681861 | -0.01435 | 0.1565 | 0.8887 | 2.88E-08 |
| 45 | rs11694904 | 0.01215 | 0.3384 | 0.6451 | 4.78E-11 |
| 46 | rs11732657 | -0.01274 | 0.7007 | 0.5623 | 9.54E-11 |
| 47 | rs117468730 | -0.03521 | 0.0119 | 0.578599 | 3.79E-09 |
| 48 | rs11752914 | -0.01208 | 0.1973 | 0.5447 | 2.13E-08 |
| 49 | rs11772580 | -0.01199 | 0.2534 | 0.729699 | 2.57E-09 |
| 50 | rs11871429 | -0.01425 | 0.2041 | 0.2291 | 1.92E-12 |
| 51 | rs12028010 | -0.01696 | 0.2228 | 0.676401 | 4.51E-17 |
| 54 | rs12375949 | 0.01447 | 0.5697 | 0.3014 | 3.31E-17 |
| 55 | rs12468040 | -0.01432 | 0.6037 | 0.1274 | 2.46E-16 |
| 56 | rs12503522 | -0.01125 | 0.2483 | 0.4217 | 2.24E-09 |
| 57 | rs12519073 | -0.01221 | 0.2381 | 0.7173 | 1.60E-09 |
| 58 | rs12574281 | 0.01077 | 0.3997 | 0.1654 | 8.85E-10 |
| 59 | rs12602286 | 0.01701 | 0.8861 | 0.3944 | 2.37E-11 |
| 60 | rs12643771 | 0.01518 | 0.3112 | 0.9828 | 1.61E-16 |
| 61 | rs12682775 | 0.01187 | 0.2143 | 0.1208 | 5.99E-09 |
| 62 | rs12804787 | -0.01814 | 0.06803 | 0.3469 | 2.96E-08 |
| 63 | rs1291818 | -0.01085 | 0.5153 | 0.3922 | 1.78E-10 |
| 64 | rs12940014 | 0.00936 | 0.5204 | 0.6622 | 3.73E-08 |
| 66 | rs13010566 | 0.0106 | 0.5612 | 0.08074 | 4.59E-10 |
| 67 | rs13029509 | -0.01049 | 0.4677 | 0.7901 | 7.17E-10 |
| 68 | rs13090388 | 0.02852 | 0.3095 | 0.03809 | 4.29E-54 |
| 70 | rs13141210 | 0.01361 | 0.5085 | 0.8978 | 2.26E-15 |
| 71 | rs13145650 | -0.01918 | 0.90816 | 0.6849 | 3.80E-10 |
| 72 | rs1334297 | 0.02449 | 0.784 | 0.2238 | 3.06E-37 |
| 73 | rs13422673 | -0.01201 | 0.4847 | 0.08805 | 1.74E-12 |
| 74 | rs1363862 | -0.01171 | 0.2602 | 0.1169 | 1.02E-09 |
| 75 | rs1381247 | -0.01013 | 0.2908 | 0.773101 | 2.46E-08 |
| 76 | rs1391438 | -0.0167 | 0.6854 | 0.9469 | 5.79E-20 |
| 77 | rs1427298 | 0.0102 | 0.4116 | 0.9858 | 3.28E-09 |
| 78 | rs1450782 | -0.00945 | 0.602 | 0.9502 | 4.93E-08 |
| 80 | rs152603 | 0.01019 | 0.3861 | 0.9777 | 9.47E-09 |
| 81 | rs1558727 | -0.01069 | 0.4847 | 0.497 | 3.09E-10 |
| 82 | rs1566085 | 0.01645 | 0.5697 | 0.4966 | 6.90E-22 |
| 83 | rs1569092 | 0.01807 | 0.182 | 0.4106 | 1.16E-14 |
| 84 | rs1584469 | -0.01303 | 0.3112 | 0.9756 | 2.10E-12 |
| 86 | rs1595973 | -0.01002 | 0.5595 | 0.634501 | 6.56E-09 |
| 87 | rs1618725 | 0.01477 | 0.5204 | 0.725401 | 2.22E-17 |
| 88 | rs1620977 | -0.02046 | 0.6905 | 0.1432 | 1.14E-25 |
| 89 | rs1671770 | -0.01342 | 0.8061 | 0.9162 | 1.91E-09 |
| 90 | rs16846463 | -0.02256 | 0.1173 | 0.9572 | 1.38E-15 |
| 91 | rs16854920 | 0.01007 | 0.3554 | 0.3233 | 2.51E-08 |
| 93 | rs16995054 | -0.0139 | 0.2007 | 0.5798 | 2.52E-11 |
| 94 | rs17048855 | 0.01184 | 0.3248 | 0.3342 | 3.27E-11 |
| 95 | rs17110109 | 0.01023 | 0.3776 | 0.07002 | 4.71E-09 |
| 96 | rs17126938 | 0.01536 | 0.1207 | 0.3572 | 8.14E-10 |
| 97 | rs17425572 | -0.01224 | 0.5425 | 0.7984 | 6.89E-13 |
| 98 | rs17489649 | -0.0139 | 0.3265 | 0.654501 | 1.57E-14 |
| 100 | rs17551064 | -0.01493 | 0.1599 | 0.3139 | 8.62E-11 |
| 101 | rs17563464 | -0.01477 | 0.2041 | 0.878 | 2.89E-12 |
| 102 | rs17565975 | -0.01142 | 0.5306 | 0.2025 | 2.56E-11 |
| 103 | rs17598675 | 0.01199 | 0.5187 | 0.7595 | 1.75E-12 |
| 104 | rs176218 | 0.01883 | 0.2007 | 0.3597 | 1.85E-18 |
| 105 | rs1827540 | -0.0106 | 0.466 | 0.751299 | 4.50E-10 |
| 106 | rs1866823 | 0.01009 | 0.551 | 0.347 | 3.81E-09 |
| 108 | rs192436652 | -0.03497 | 0.02211 | 0.07452 | 1.35E-10 |
| 109 | rs1925576 | 0.00997 | 0.4422 | 0.968 | 4.94E-09 |
| 110 | rs1947114 | 0.01071 | 0.2551 | 0.549 | 2.64E-08 |
| 111 | rs1964927 | -0.01423 | 0.6378 | 0.02649 | 9.90E-16 |
| 112 | rs2052285 | 0.01123 | 0.5765 | 0.2852 | 1.34E-10 |
| 113 | rs2067854 | 0.01477 | 0.182 | 0.1581 | 1.38E-12 |
| 114 | rs2179152 | 0.01455 | 0.6429 | 0.4688 | 1.21E-16 |
| 115 | rs2182505 | -0.01086 | 0.7364 | 0.5756 | 1.64E-08 |
| 116 | rs225291 | -0.01205 | 0.8027 | 0.1725 | 1.84E-08 |
| 117 | rs2256965 | -0.01128 | 0.5425 | 0.01791 | 1.59E-10 |
| 118 | rs2283076 | -0.01143 | 0.2143 | 0.691801 | 2.07E-08 |
| 119 | rs2287838 | -0.01152 | 0.534 | 0.4878 | 1.53E-11 |
| 120 | rs2302761 | 0.01354 | 0.1905 | 0.8533 | 1.00E-10 |
| 121 | rs2347526 | 0.01395 | 0.6378 | 0.278 | 6.84E-15 |
| 123 | rs242093 | -0.01031 | 0.5476 | 0.783401 | 2.07E-09 |
| 124 | rs2447535 | 0.01181 | 0.7245 | 0.544 | 1.69E-10 |
| 127 | rs2554835 | 0.00974 | 0.398 | 0.2788 | 2.69E-08 |
| 128 | rs2570497 | -0.01233 | 0.6735 | 0.7877 | 3.03E-12 |
| 129 | rs2725370 | 0.01536 | 0.7109 | 0.4882 | 1.97E-16 |
| 130 | rs277828 | -0.01091 | 0.2568 | 0.9801 | 2.71E-08 |
| 131 | rs2787101 | 0.00968 | 0.6173 | 0.9534 | 2.50E-08 |
| 132 | rs2819336 | -0.01828 | 0.6616 | 0.2414 | 5.46E-25 |
| 133 | rs2820314 | -0.011 | 0.3163 | 0.126 | 9.34E-10 |
| 135 | rs28513670 | 0.01477 | 0.1531 | 0.3558 | 5.06E-11 |
| 136 | rs2885198 | -0.01025 | 0.5017 | 0.6577 | 1.81E-09 |
| 137 | rs2901616 | 0.00941 | 0.517 | 0.3936 | 3.77E-08 |
| 138 | rs2905426 | 0.01037 | 0.6446 | 0.6137 | 9.26E-09 |
| 140 | rs2971970 | 0.01654 | 0.7721 | 0.448 | 1.25E-15 |
| 141 | rs2998315 | 0.01269 | 0.5782 | 0.1049 | 1.12E-13 |
| 142 | rs3013014 | -0.01024 | 0.6105 | 0.5166 | 2.92E-09 |
| 143 | rs301800 | -0.01516 | 0.8197 | 0.4947 | 1.33E-11 |
| 144 | rs3026996 | -0.01537 | 0.284 | 0.877 | 1.05E-14 |
| 145 | rs31940 | 0.01548 | 0.1344 | 0.4188 | 3.24E-10 |
| 147 | rs337637 | 0.01123 | 0.3367 | 0.760901 | 2.11E-10 |
| 148 | rs34316 | -0.02016 | 0.5799 | 0.7696 | 3.35E-30 |
| 149 | rs34394051 | 0.01392 | 0.1599 | 0.7799 | 6.20E-09 |
| 150 | rs34485537 | 0.01075 | 0.3895 | 0.5612 | 5.67E-10 |
| 152 | rs35039375 | -0.01983 | 0.09354 | 0.308 | 1.22E-11 |
| 153 | rs35309068 | 0.01321 | 0.466 | 0.09649 | 1.15E-14 |
| 154 | rs35316276 | 0.01173 | 0.2942 | 0.3414 | 1.52E-09 |
| 155 | rs35417702 | -0.01445 | 0.5765 | 0.385 | 1.93E-17 |
| 156 | rs35475880 | -0.01511 | 0.1837 | 0.548001 | 3.80E-13 |
| 158 | rs36083520 | 0.01629 | 0.1667 | 0.2309 | 2.60E-13 |
| 159 | rs36119825 | 0.01063 | 0.4694 | 0.994 | 4.82E-10 |
| 160 | rs363096 | 0.01363 | 0.5748 | 0.7293 | 2.04E-15 |
| 162 | rs3788556 | -0.01138 | 0.5408 | 0.4172 | 2.78E-11 |
| 164 | rs3809634 | 0.01058 | 0.335 | 0.48 | 1.09E-08 |
| 165 | rs3890802 | -0.01133 | 0.2687 | 0.8984 | 2.74E-09 |
| 166 | rs3897821 | -0.01502 | 0.3503 | 0.3029 | 8.25E-17 |
| 169 | rs4073894 | 0.01524 | 0.1769 | 0.04867 | 5.40E-13 |
| 170 | rs4328757 | 0.01067 | 0.6514 | 0.7968 | 9.39E-10 |
| 171 | rs4352658 | -0.0212 | 0.09014 | 0.4029 | 5.55E-12 |
| 172 | rs4369924 | 0.01362 | 0.1684 | 0.610401 | 5.82E-09 |
| 173 | rs4382592 | 0.01636 | 0.699 | 0.9496 | 1.01E-18 |
| 174 | rs4384309 | 0.0109 | 0.4796 | 0.6447 | 2.52E-10 |
| 175 | rs4392737 | -0.0097 | 0.3827 | 0.4872 | 2.24E-08 |
| 176 | rs4442732 | -0.01063 | 0.5969 | 0.8012 | 1.49E-09 |
| 177 | rs4667025 | 0.00957 | 0.3878 | 0.8705 | 3.85E-08 |
| 178 | rs4700393 | 0.02086 | 0.5289 | 0.5534 | 1.51E-34 |
| 179 | rs4726070 | 0.01251 | 0.6207 | 0.599799 | 5.95E-13 |
| 183 | rs4778058 | 0.01017 | 0.5221 | 0.832 | 2.40E-09 |
| 184 | rs4787457 | -0.01741 | 0.3146 | 0.639699 | 3.73E-23 |
| 185 | rs4810227 | 0.01272 | 0.6344 | 0.3238 | 3.57E-13 |
| 186 | rs4839155 | -0.01251 | 0.25 | 0.401 | 3.94E-10 |
| 187 | rs4846724 | 0.01018 | 0.4915 | 0.1347 | 2.26E-09 |
| 189 | rs4888746 | -0.00952 | 0.3776 | 0.7259 | 4.15E-08 |
| 190 | rs4904523 | -0.00936 | 0.4592 | 0.7971 | 3.71E-08 |
| 191 | rs4945424 | -0.00992 | 0.415 | 0.3973 | 6.94E-09 |
| 192 | rs4964046 | 0.01053 | 0.335 | 0.9681 | 3.36E-09 |
| 193 | rs4972400 | 0.01156 | 0.352 | 0.04586 | 1.70E-10 |
| 194 | rs4984541 | 0.01233 | 0.2415 | 0.9187 | 2.77E-09 |
| 196 | rs535307 | -0.01004 | 0.6769 | 0.1421 | 4.73E-08 |
| 199 | rs56391344 | 0.01571 | 0.2381 | 0.6738 | 1.34E-15 |
| 200 | rs575113 | 0.01285 | 0.2772 | 0.552199 | 5.30E-12 |
| 201 | rs59123361 | -0.02094 | 0.1054 | 0.7147 | 5.87E-13 |
| 204 | rs6122735 | 0.0105 | 0.4133 | 0.760801 | 1.49E-09 |
| 205 | rs6123924 | -0.01528 | 0.1599 | 0.7603 | 7.55E-11 |
| 206 | rs613872 | -0.0175 | 0.8282 | 0.1903 | 1.20E-14 |
| 207 | rs62097985 | -0.01288 | 0.4116 | 0.9627 | 6.06E-14 |
| 208 | rs62157915 | 0.02091 | 0.05952 | 0.955 | 1.96E-09 |
| 210 | rs62183776 | -0.01308 | 0.1905 | 0.640801 | 1.65E-09 |
| 211 | rs62184480 | -0.01528 | 0.2449 | 0.051951 | 1.28E-15 |
| 212 | rs622169 | 0.00999 | 0.4677 | 0.2382 | 1.89E-08 |
| 213 | rs62439690 | -0.01087 | 0.267 | 0.653 | 2.18E-08 |
| 214 | rs62444881 | 0.01815 | 0.1905 | 0.2408 | 5.79E-17 |
| 215 | rs6493265 | -0.01385 | 0.3895 | 0.6356 | 1.70E-15 |
| 216 | rs6513959 | -0.01177 | 0.2789 | 0.03022 | 1.88E-10 |
| 217 | rs6557171 | 0.01567 | 0.7245 | 0.3485 | 4.15E-18 |
| 219 | rs66568921 | 0.01565 | 0.3639 | 0.9429 | 7.49E-18 |
| 220 | rs6731373 | -0.01256 | 0.3367 | 0.2028 | 3.47E-12 |
| 222 | rs67885444 | 0.01406 | 0.1718 | 0.071271 | 1.48E-09 |
| 223 | rs67890737 | -0.01141 | 0.3265 | 0.8122 | 2.01E-10 |
| 224 | rs6803651 | 0.01131 | 0.415 | 0.8435 | 4.36E-11 |
| 225 | rs6805241 | -0.01413 | 0.1973 | 0.783401 | 3.09E-12 |
| 227 | rs6938002 | -0.01008 | 0.3963 | 0.3576 | 5.41E-09 |
| 228 | rs6959891 | -0.01136 | 0.2959 | 0.2688 | 1.74E-09 |
| 229 | rs7012546 | 0.01009 | 0.4201 | 0.508 | 4.93E-09 |
| 231 | rs702606 | -0.01427 | 0.1701 | 0.2501 | 1.12E-08 |
| 232 | rs7029718 | 0.02439 | 0.4354 | 0.3768 | 1.85E-44 |
| 233 | rs7031698 | 0.01248 | 0.7755 | 0.721499 | 1.26E-09 |
| 234 | rs710629 | 0.01053 | 0.6565 | 0.8615 | 2.96E-09 |
| 235 | rs71646142 | 0.01286 | 0.1735 | 0.9101 | 3.11E-09 |
| 236 | rs7233920 | -0.01315 | 0.216 | 0.08601 | 7.13E-11 |
| 237 | rs7257460 | -0.01145 | 0.2704 | 0.7624 | 1.25E-09 |
| 239 | rs72807818 | 0.01915 | 0.1241 | 0.5211 | 2.79E-14 |
| 240 | rs72828517 | 0.01836 | 0.1412 | 0.4504 | 2.83E-16 |
| 241 | rs72840994 | 0.01247 | 0.182 | 0.710799 | 7.77E-09 |
| 242 | rs730384 | 0.01016 | 0.4558 | 0.1672 | 3.01E-09 |
| 244 | rs7321274 | -0.01275 | 0.1956 | 0.5399 | 1.59E-09 |
| 245 | rs73301698 | -0.01291 | 0.2262 | 0.006559 | 5.81E-10 |
| 246 | rs7332724 | -0.01149 | 0.2619 | 0.6192 | 1.26E-09 |
| 247 | rs73344830 | -0.0172 | 0.602 | 0.6375 | 1.95E-23 |
| 248 | rs736282 | -0.01082 | 0.5153 | 0.3593 | 2.07E-10 |
| 249 | rs73874335 | -0.0199 | 0.05952 | 0.3268 | 3.40E-08 |
| 250 | rs743316 | -0.01185 | 0.182 | 0.4173 | 1.20E-08 |
| 251 | rs74643044 | 0.02323 | 0.02721 | 0.631801 | 1.80E-09 |
| 252 | rs74701752 | 0.01591 | 0.09524 | 0.772499 | 2.38E-08 |
| 253 | rs7481514 | 0.01072 | 0.665 | 0.2615 | 1.56E-09 |
| 254 | rs74998289 | -0.01821 | 0.2398 | 0.1405 | 1.31E-17 |
| 255 | rs7594904 | 0.00969 | 0.4184 | 0.4854 | 2.05E-08 |
| 256 | rs7603132 | 0.01317 | 0.1548 | 0.1066 | 9.17E-10 |
| 257 | rs76076331 | 0.01873 | 0.131 | 0.1454 | 4.40E-14 |
| 258 | rs7650602 | 0.00939 | 0.4286 | 0.5705 | 4.11E-08 |
| 259 | rs76608582 | 0.02798 | 0.04082 | 0.08154 | 3.11E-10 |
| 261 | rs77025239 | -0.01422 | 0.1088 | 0.2613 | 1.33E-09 |
| 262 | rs77128898 | -0.02769 | 0.02211 | 0.4229 | 9.47E-09 |
| 263 | rs77702622 | -0.02447 | 0.07653 | 0.089 | 2.99E-12 |
| 265 | rs77835879 | -0.01601 | 0.09014 | 0.9239 | 2.68E-08 |
| 266 | rs7796203 | -0.01074 | 0.5255 | 0.9479 | 3.60E-10 |
| 267 | rs7803932 | 0.0143 | 0.1565 | 0.9369 | 2.44E-10 |
| 268 | rs7808399 | 0.0107 | 0.5476 | 0.9874 | 3.78E-10 |
| 270 | rs7863447 | 0.01678 | 0.8333 | 0.06842 | 5.91E-13 |
| 271 | rs78721320 | 0.01307 | 0.2041 | 0.6728 | 2.28E-09 |
| 272 | rs790647 | -0.01482 | 0.2347 | 0.383 | 2.17E-13 |
| 274 | rs7924036 | 0.01501 | 0.5391 | 0.5142 | 1.07E-18 |
| 275 | rs79265434 | 0.02331 | 0.1173 | 0.8752 | 6.08E-19 |
| 276 | rs79269403 | 0.01447 | 0.2228 | 0.8916 | 1.17E-12 |
| 278 | rs795230 | 0.00952 | 0.4184 | 0.3469 | 2.97E-08 |
| 279 | rs79523955 | -0.01802 | 0.09524 | 0.8739 | 1.87E-10 |
| 280 | rs7977614 | 0.01325 | 0.3078 | 0.006303 | 2.09E-11 |
| 281 | rs7993663 | 0.0118 | 0.3571 | 0.528 | 3.25E-11 |
| 282 | rs8008382 | 0.01208 | 0.6871 | 0.8077 | 6.12E-11 |
| 283 | rs80171383 | 0.0145 | 0.1241 | 0.4221 | 1.83E-09 |
| 284 | rs8020034 | 0.01782 | 0.2058 | 0.5152 | 1.17E-15 |
| 285 | rs818415 | 0.01235 | 0.182 | 0.5158 | 1.72E-08 |
| 286 | rs837080 | 0.01092 | 0.4932 | 0.3626 | 1.43E-10 |
| 287 | rs892612 | 0.01464 | 0.8418 | 0.7088 | 6.63E-10 |
| 288 | rs894067 | 0.01041 | 0.3929 | 0.01982 | 2.74E-09 |
| 289 | rs9289300 | 0.01512 | 0.1837 | 0.8758 | 1.10E-10 |
| 290 | rs9320493 | -0.01394 | 0.8639 | 0.01739 | 6.13E-09 |
| 291 | rs9342482 | 0.01264 | 0.2908 | 0.6704 | 1.36E-10 |
| 292 | rs9349956 | 0.01881 | 0.2398 | 0.717 | 6.28E-17 |
| 293 | rs9372625 | 0.02383 | 0.4133 | 0.7486 | 6.76E-42 |
| 294 | rs9384679 | -0.00959 | 0.4082 | 0.6707 | 4.88E-08 |
| 295 | rs9386319 | 0.00991 | 0.4269 | 0.8097 | 1.27E-08 |
| 296 | rs9436866 | 0.01882 | 0.09524 | 0.01621 | 7.45E-11 |
| 297 | rs9503598 | 0.01079 | 0.4388 | 0.2501 | 3.12E-10 |
| 299 | rs9556958 | -0.0108 | 0.5289 | 0.749899 | 2.38E-10 |
| 300 | rs9616906 | 0.01497 | 0.4235 | 0.8885 | 2.92E-18 |
| 301 | rs9679654 | 0.01042 | 0.4847 | 0.1066 | 1.29E-09 |
| 303 | rs9704097 | -0.0103 | 0.4728 | 0.778501 | 1.61E-09 |
| 304 | rs9882532 | -0.01208 | 0.3639 | 0.127 | 8.17E-12 |
| 305 | rs9914918 | 0.01155 | 0.2823 | 0.007201 | 8.90E-10 |
| 306 | rs9933256 | -0.01134 | 0.4082 | 0.000348 | 4.57E-11 |
| 307 | rs9936270 | -0.0136 | 0.3078 | 0.2223 | 6.43E-12 |
| 309 | rs9964724 | 0.01978 | 0.6599 | 0.4094 | 2.66E-27 |
| 310 | rs9995567 | 0.00998 | 0.3827 | 0.8612 | 1.93E-08 |

**Supplemental Table 15.** SNP list used in the MR analysis of educational attainment and Kidney disease.

| **id.exposure** | **exposure** | **outcome** | **nsnp** | **b** | **OR** | **pval** |
| --- | --- | --- | --- | --- | --- | --- |
| ebi-a-GCST006572 | Cognitive performance | Kidney disease | 78 | 0.02893336 | 1.0293560 | 0.84627987 |
| ieu-a-1239 | Years of schooling | Kidney disease | 267 | -0.36261011 | 0.6958577 | 0.02366585 |

**Supplemental Figure 1.** Leave-one-out results of BMI, WHR, smoking heaviness and SBP on KD.


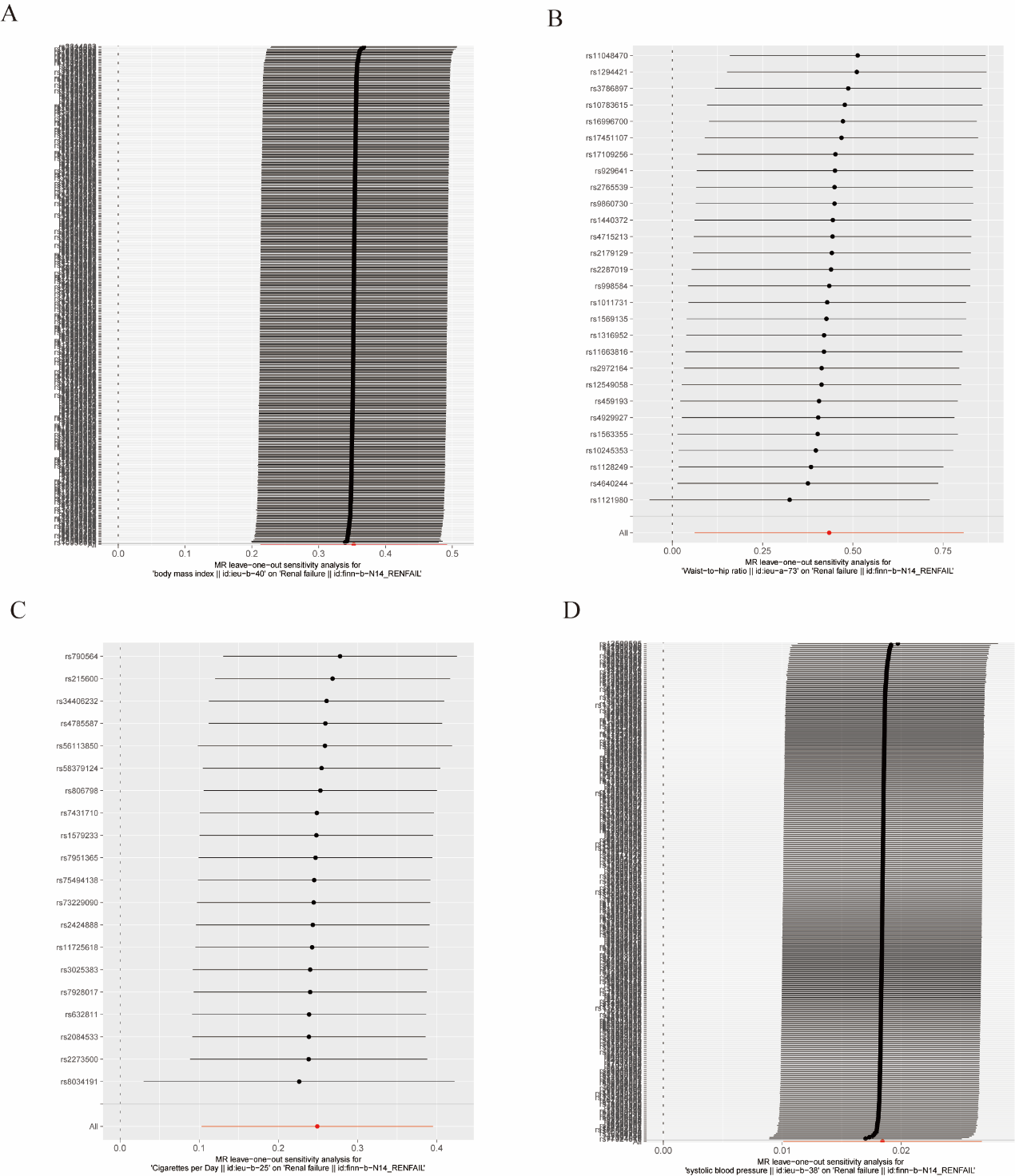


A: Forest plot leave-one-out analysis of BMI on KD; B: Forest plot leave-one-out analysis of WHR on KD; C: Forest plot leave-one-out analysis of smoking heaviness on KD; D: Forest plot leave-one-out analysis of SBP on KD;BMI, body mass index; WHR, waist-to-hip ratio; SBP, systolic blood pressure; KD: Kidney disease.
